# Human Cytomegalovirus Infection in Haematopoietic Stem Cell Transplant Recipients and CAR T Cell Recipients *– PART 1: Risk Factors, Clinical Impact and Immune Response*

**DOI:** 10.1002/rmv.70142

**Published:** 2026-04-14

**Authors:** Danya Kaplan, Emily Blyth, Gaurav Sutrave, Michelle K. Yong, David J. Gottlieb, Allison Abendroth, Barry Slobedman, Lauren Stern

**Affiliations:** ^1^ Infection, Immunity, and Inflammation School of Medical Sciences Faculty of Medicine and Health The University of Sydney Sydney New South Wales Australia; ^2^ Charles Perkins Centre the Sydney Institute for Infectious Diseases The University of Sydney Sydney New South Wales Australia; ^3^ Westmead Institute for Medical Research Westmead New South Wales Australia; ^4^ Department of Haematology Westmead Hospital Blood Transplant and Cell Therapies Program Sydney New South Wales Australia; ^5^ Faculty of Medicine and Health Sydney Medical School The University of Sydney Sydney New South Wales Australia; ^6^ Department of Infectious Diseases Peter MacCallum Cancer Centre Melbourne Victoria Australia; ^7^ Sir Peter MacCallum Department of Oncology The University of Melbourne Melbourne Victoria Australia; ^8^ Department of Infectious Diseases Royal Melbourne Hospital Melbourne Victoria Australia; ^9^ National Centre for Infections in Cancer Peter MacCallum Cancer Centre Melbourne Victoria Australia

**Keywords:** CAR T, chimeric antigen receptor, CMV, cytomegalovirus, haematopoietic stem cell transplant, HSCT

## Abstract

Human cytomegalovirus (HCMV) is one of the most important opportunistic pathogens in immunocompromised individuals, including allogeneic haematopoietic stem cell transplant (allo‐HSCT) recipients. In allo‐HSCT, HCMV seropositivity of the recipient and donor is associated with inferior survival outcomes, and post‐transplant HCMV reactivation is a frequent complication, necessitating close viral monitoring and pre‐emptive and prophylactic antiviral therapies. We present a review in two parts that focuses on the risk factors, immunological responses and treatment strategies for HCMV infection in allo‐HSCT recipients, and also explores current evidence surrounding HCMV reactivation in recipients of chimeric antigen receptor T cell (CAR T) therapies. In the current article (Part 1), the impact of HCMV infection in allo‐HSCT and CAR T cell recipients is investigated. HCMV reactivation in allo‐HSCT recipients is associated with increased mortality, graft‐versus‐host disease (GvHD) and other microbial infections. Prominent alterations in T cell and natural killer (NK) cell recovery represent distinct immune reconstitution features associated with HCMV reactivation. Immunological biomarkers to predict HCMV complications have been proposed and their adoption in future immune monitoring strategies may allow individualised risk assessment to guide antiviral treatment decisions. The clinical significance of HCMV reactivation after CAR T cell infusion is yet to be fully determined. Continued viral surveillance and investigation of viral dynamics with correlative studies of immune function are needed in this patient population. Current and emerging strategies for treatment and prevention of HCMV complications in allo‐HSCT, including use of letermovir prophylaxis and adoptive HCMV‐specific T cell therapies, are explored in the following article (Part 2).

**Abbreviations**
Allo‐HSCT: Allogeneic haematopoietic stem cell transplant
BAL: Bronchoalveolar lavage
CAR: Chimeric antigen receptor
CAR T: Chimeric antigen receptor T cells
COVID‐19: Coronavirus disease 2019
CRS: Cytokine release syndrome
csHCMVi: Clinically significant HCMV infection
D+: HCMV seropositive donor
D−: HCMV seronegative donor
ELISA: Enzyme linked immunosorbent assay
ELISpot: Enzyme‐linked immunospot
FCRL6: Fc receptor‐like 6
GvHD: Graft‐versus‐host disease
HCMV: Human cytomegalovirus
HHV‐5: human herpesvirus 5
HLA: Human leukocyte antigen
HSCT: Haematopoietic stem cell transplant
ICAHT: Immune effector cell associated haematological toxicity
ICANS: Immune associated neurotoxicity syndrome
IFN‐γ: Interferon‐γ
IL‐2: Interleukin‐2
iNKT: Invariant natural killer T
IU: International units
IVIG: Intravenous immunoglobulin
MAIT: Mucosal‐associated invariant T
MCMV: Murine cytomegalovirus
NK: Natural killer
pp65: phosphoprotein 65
PTCy: Post‐transplant cyclophosphamide
R+: HCMV seropositive recipient
R−: HCMV seronegative recipient
TCR: T cell receptor
Treg: Regulatory T cell
WHO: World Health Organisation

## 1 Introduction

Allogeneic haematopoietic stem cell transplantation (allo‐HSCT) is a therapy with curative potential for high‐risk haematological malignancies and some non‐malignant diseases, including bone‐marrow failure syndromes, primary immunodeficiencies and congenital metabolic disorders. Human cytomegalovirus (HCMV) is a major opportunistic pathogen in allo‐HSCT recipients, and requires close virological surveillance and management with pre‐emptive or prophylactic antiviral drug therapies to limit viral replication and prevent end‐organ disease. Higher levels of HCMV DNAemia in the first 100 days post‐transplant are associated with increased early overall mortality (< day 60 post‐transplant) and non‐relapse mortality [1], and outcomes of treatment resistant/refractory HCMV infection and end‐organ disease remain poor. Recent developments in transplant practices, including the introduction of letermovir for HCMV primary prophylaxis, are changing the epidemiological and immunological landscape of HCMV reactivation after allo‐HSCT. In addition, evidence demonstrating the significance of HCMV reactivation in new patient populations such as chimeric antigen receptor T cell (CAR T) therapy recipients is emerging.

This review article is presented in two parts. Part 1 (the current article) explores the risk factors and immune responses associated with HCMV reactivation after allo‐HSCT, and evaluates the incidence and impact of HCMV infection in CAR T recipients. Treatment strategies for HCMV reactivation and disease in allo‐HSCT recipients, specifically antiviral drug therapies and adoptive HCMV‐specific T cell therapy approaches, are discussed in ‘*PART 2: Antiviral Therapy and Virus‐Specific T cell Therapy for HCMV in Allo‐HSCT’* [2].

## 2 Human Cytomegalovirus

HCMV, or human herpesvirus 5 (HHV‐5), is a large, species‐specific double‐stranded DNA virus of the Betaherpesvirinae subfamily, and has an estimated global seroprevalence of 83% in the general population [3]. HCMV is transmitted through direct contact with infectious bodily fluids, via the placenta to the developing foetus, and through allografts and non‐leukodepleted blood products from HCMV seropositive donors [4, 5, 6]. In healthy, immunocompetent individuals, HCMV replication is typically well‐controlled by robust host immune responses, and primary HCMV infection, reinfection or reactivation episodes usually occur without overt symptoms or disease. In contrast, HCMV infection or reactivation in immunocompromised individuals can cause severe disease and mortality.

HCMV persists in the host following initial infection and establishes life‐long latency in CD34^+^ haematopoietic stem cells and myeloid progenitor cells in the bone marrow [7, 8], with periodic episodes of reactivation. During latency, no replicative virus is produced and the viral genome is retained as an episome, with limited viral gene expression [9, 10]. The latent viral genome is carried in immature myeloid lineage cells, and CD14^+^ monocytes in peripheral blood [11]. In addition to the myeloid lineage, there may also be other, less well‐defined, cellular reservoirs of HCMV latency or persistence within tissues [12]. The terminal differentiation of latently‐infected myeloid cells to mature macrophages or dendritic cells [13, 14] can trigger HCMV reactivation, which results in renewed viral replication and is enhanced by inflammatory signals [14, 15, 16].

## 3 HCMV Reactivation in Allo‐HSCT Recipients

HCMV reactivation is one of the most common and clinically significant viral complications in both adult and paediatric allo‐HSCT recipients. Individuals undergoing allo‐HSCT are subject to profoundly immunosuppressive transplant conditioning regimens and experience a significant post‐transplant immune deficiency while donor‐derived immunohaematopoietic reconstitution takes place [17]. Without antiviral prophylaxis, HCMV reactivation is commonly detected in the first 100 days post‐transplant [1, 18], and can derive from endogenous virus present in a HCMV seropositive recipient, as well as primary transmission through a graft from a seropositive donor.

HCMV is able to productively replicate in the parenchymal and stromal cell types of most organs [19]. Uncontrolled HCMV replication in allo‐HSCT recipients can result in the development of end‐organ diseases such as pneumonitis, gastrointestinal disease, encephalitis, hepatitis and retinitis. The adoption of pre‐emptive/prophylactic antiviral therapies and sensitive viral surveillance methods has resulted in a marked reduction in HCMV end‐organ disease (approximately 2%–5% incidence [1, 20, 21], but higher in some cohorts [22]). Nonetheless, when HCMV end‐organ disease develops it can cause significant morbidity and be life‐threatening, with mortality for HCMV pneumonia remaining high (30%) [23].

HCMV is measured in blood and body tissues using sensitive qualitative and quantitative assays. Detection of phosphoprotein 65 (pp65) antigenemia has generally fallen out of use in favour of more sensitive nucleic acid detection methods that target one to two viral genes [24, 25]. The performance of commercial kits and in house assays in quantitation can vary substantially so a World Health Organisation (WHO) standard is used to calibrate reagents and standard operating procedures with viral load expressed as international units (IU) per mL [26]. Despite this standard there remains some variation between and within laboratories, so some caution should be exercised when interpreting absolute viral copy number [27]. Viral load in whole blood can be 0.5‐1 logs higher than plasma due to the former including cell associated and cell free virus, highlighting the importance of consistency when monitoring longitudinally [28, 29].

Surveillance for HCMV reactivation is typically conducted by weekly quantitative HCMV DNAemia testing in peripheral blood in the first 100 days post‐transplant. Clinically‐significant HCMV infection (csHCMVi) is defined as HCMV end‐organ disease, or HCMV viraemia that leads to the initiation of pre‐emptive antiviral therapy at pre‐specified viral load thresholds [30]. There is no consensus on HCMV viral load threshold levels for starting pre‐emptive therapy due to assay variability and patient heterogeneity. A viral load threshold of 2‐3 log_10_ IU/mL in plasma is commonly used and may be optimal for pre‐emptive therapy commencement, with patient's underlying risk and the pace of rise being taken into consideration [21, 31].

HCMV DNAemia is an independent risk factor for non‐relapse mortality after allo‐HSCT [1, 32], although HCMV end‐organ disease itself accounts for only a small proportion of non‐relapse deaths [1]. HCMV reactivation and disease are associated with increased risk of early and late invasive fungal infections [33, 34, 35] and graft‐versus‐host disease (GvHD) [36, 37]. A higher incidence of mortality due to bacterial and fungal infections is observed in D+/R− patients compared to D−/R− patients [38].

Underlying the association of HCMV reactivation with poor transplant outcomes and other infectious complications post‐HSCT may be shared risk factors such as poor immune reconstitution or the intensity of immunosuppression (e.g., use of corticosteroids). However, it is possible that direct effects of HCMV replication and disease, as well as indirect impacts of HCMV on the host [39], such as potential toxicities from anti‐HCMV drugs (e.g., ganciclovir, foscarnet) and HCMV‐mediated modulation of immune recovery and innate and adaptive immune responses, play a role. One approach by which HCMV can influence host immunity is through the expression of multiple viral immunomodulatory gene products [40]. These virally‐encoded functions include molecular mimicry of human cytokines/chemokines and receptors [41], inhibition of antigen presentation [42, 43], evasion of natural killer (NK) cell responses [44], and impairment of monocyte, dendritic cell and macrophage functions [45, 46, 47].

## 4 Impact of HCMV Serostatus on HCMV Reactivation Risk and Transplant Outcomes

The combination of pre‐transplant recipient and donor IgG HCMV serostatus is a major risk determinant for HCMV reactivation after allo‐HSCT [48]. Approximately 60%–70% of HCMV seropositive recipients develop HCMV reactivation after allo‐HSCT without antiviral prophylaxis [20, 49]. HCMV seropositive recipients of seronegative donors (D−/R+) are at the highest risk of HCMV reactivation and disease, as seronegative donor grafts lack pre‐existing HCMV‐specific immune cells which may provide early protection against viral replication [50]. The incidence of HCMV reactivation in a HCMV seronegative recipient with a seropositive donor (D+/R−) after allo‐HSCT is approximately 10% [48, 51].

Studies from the pre‐letermovir era indicate that pre‐transplant HCMV seropositivity of the recipient and donor are associated with inferior survival outcomes post‐HSCT [52, 53]. In particular, recipient HCMV seropositivity is linked with poor transplant outcomes and increased overall and non‐relapse mortality [52, 54]. Recipient HCMV seropositivity was also associated with lower overall survival in allo‐HSCT recipients diagnosed with COVID‐19 during the pandemic [55].

HCMV seropositivity in the recipient may be less prognostic when letermovir is used for prophylaxis, as recent evidence has shown a mortality benefit associated with receipt of letermovir prophylaxis in HCMV seropositive recipients [56, 57]. Letermovir prophylaxis results in a lower incidence of csHCMVi [20] and potentially reduces exposure to antiviral agents used in pre‐emptive therapy such as ganciclovir and foscarnet, which can cause serious toxicities including myelosuppression and nephrotoxicity (see *PART 2: Antiviral Therapy and Virus‐Specific T Cell Therapy for HCMV in Allo‐HSCT*).

Selection of a HCMV seropositive donor for a seropositive recipient is associated with improved survival in the setting of unrelated donor allo‐HSCT with use of myeloablative conditioning, compared to use of a seronegative donor [58]. In matched unrelated paediatric HSCT, a HCMV seropositive donor for a seropositive recipient was independently associated with improved survival outcomes compared with a HCMV seronegative donor [59]. In HCMV seropositive recipients, the adverse impacts of older donor age and HLA mismatch on overall survival after unrelated HSCT appear to be modulated by donor HCMV serostatus [60], whereby older donor age and HLA mismatch were associated with inferior overall survival in D+/R+ patients but not D−/R+ patients in this study [60].

## 5 Risk Factors for HCMV Reactivation and Disease

**FIGURE 1:**
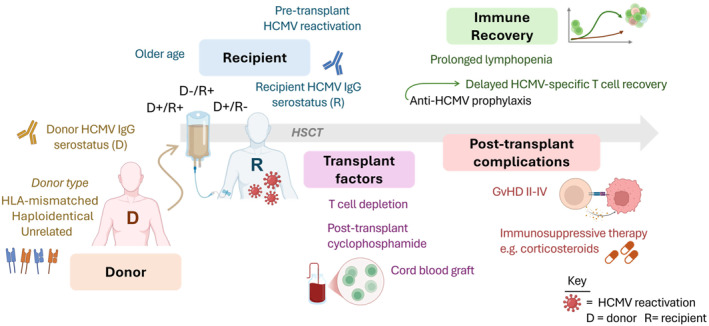
Risk factors for human cytomegalovirus (HCMV) reactivation in allogeneic haematopoietic stem cell transplant (HSCT) patients. Donor (D), recipient (R) and transplant variables are among the risk factors for HCMV reactivation and end‐organ disease after allo‐HSCT. Pre‐transplant recipient and donor HCMV serostatus is an important feature that influences the risk of reactivation. Post‐transplant complications such as graft‐versus‐host disease (GvHD) and the use of high dose immunosuppressive steroid therapies for GvHD increase the risk of clinically significant HCMV infection. Slow immune recovery, and delayed HCMV‐specific T cell reconstitution, which can result from reduced viral antigen exposure in individuals receiving antiviral prophylaxis, increase the risk of HCMV complications and late reactivation. +, HCMV seropositive; ‐, HCMV seronegative; HLA, human leukocyte antigen. Created with BioRender. Stern, L. (2026) https://BioRender.com/8cgzmv3.

The risk of HCMV reactivation and disease in allo‐HSCT recipients is influenced by factors including the combination of pre‐transplant recipient/donor HCMV serostatus, the use of unrelated, HLA‐mismatched or haploidentical donors, older recipient age, use of umbilical cord blood as the graft source, in vitro and in vivo T cell depletion (e.g., anti‐thymocyte globulin, post‐transplant cyclophosphamide [PTCy]), acute GvHD grades II‐IV and use of systemic steroids for GvHD [22, 48, 61, 62, 63, 64, 65] (**Figure** **1**). Delayed or incomplete immune reconstitution, in particular the slow recovery of HCMV‐specific CD8^+^ and CD4^+^ T cell responses, is linked to increased risk of developing csHCMVi and late HCMV reactivation (after 100 days post‐transplant) [66, 67, 68, 69, 70, 71, 72, 73, 74, 75]. Cytokine release syndrome (CRS) after haploidentical transplantation has been reported to be associated with increased risk of csHCMVi [76].

Pre‐transplant HCMV DNAemia has also been identified as a significant risk factor for post‐transplant HCMV reactivation and disease [77]. Pre‐transplant lymphopenia is among the risk factors for HCMV reactivation before allo‐HSCT [77]. Higher recipient anti‐HCMV IgG titres before transplantation have been found to correlate with increased risk of HCMV reactivation after allo‐HSCT in some studies [78, 79], suggesting quantitation of pre‐transplant anti‐HCMV IgG titres may be informative in predicting post‐transplant HCMV reactivation.

## 6 HCMV End‐Organ Disease and Late Onset HCMV Reactivation

HCMV DNAemia is often asymptomatic and while high or increasing viral loads are linked to increased risk of developing HCMV end‐organ disease [80, 81], HCMV disease, particularly in the gastrointestinal tract, can occur in a localised manner and may not be accompanied by detectable HCMV DNAemia or viraemia [82]. Pulmonary HCMV shedding can also occur in allo‐HSCT patients with and without HCMV pneumonia. Higher HCMV DNA loads (> 500 IU/mL) in bronchoalveolar lavage (BAL) fluid have been suggested to differentiate HCMV pneumonia from pulmonary shedding [83] but universal HCMV DNA load thresholds in BAL that indicate HCMV pneumonia are difficult to determine [84].

Use of antiviral prophylaxis can delay the reconstitution of HCMV‐specific T cell functional responses, increasing the risk of late HCMV reactivation after prophylaxis cessation [85, 86]. This reflects the likely importance of viral antigen exposure in stimulating the recovery of HCMV‐specific immune responses after allo‐HSCT [87]. Late‐onset HCMV disease can also occur in patients treated with prophylaxis or pre‐emptive therapy (5%–7%) [88]. Risk factors for late HCMV disease include prior HCMV viraemia, receipt of corticosteroids (≥ 1 mg/kg) after day 100 post‐HSCT, acute and chronic GvHD, lymphopenia and use of HLA‐mismatched donors [88].

## 7 HCMV and GvHD

There is a relationship between HCMV and GvHD after allo‐HSCT [32, 36]. Both acute GvHD and systemic steroids, typically used as first‐line therapy for GvHD, are important risk factors for HCMV end‐organ disease [88, 89]. HCMV gastrointestinal disease now represents the most common manifestation of HCMV disease [1, 18, 34] and can occur concurrently with gastrointestinal GvHD [88, 90]. A study by Akahoshi et al. (2022) identified a 5.7% incidence of HCMV gastroenteritis within 1 year of acute GvHD grade II‐IV diagnosis [91], and Sadowska‐Klasa et al. (2024) reported that a diagnosis of intestinal GvHD preceded gastrointestinal HCMV disease in approximately 60% of patients with late gastrointestinal HCMV disease [88]. As gastrointestinal GvHD and HCMV disease can present with similar symptoms, evidence of HCMV in tissue biopsies, such as via immunohistochemical staining, is needed for accurate diagnosis [30].

Immune dysregulation and local inflammation associated with GvHD may promote HCMV reactivation, and suppression of T cell responses arising from GvHD and its immunosuppressive therapy [87] can impair immune control of HCMV replication. The use of PTCy has emerged as an effective GvHD prophylaxis strategy, but has been associated with increased risk of infections, including HCMV reactivation, particularly in HLA‐haploidentical transplantation [18, 92, 93, 94]. HCMV reactivation in PTCy recipients is associated with a higher incidence of chronic GvHD [93], reflecting the bidirectional relationship between HCMV and GvHD [36], where HCMV reactivation has been linked to an increased risk of GvHD development [32, 36, 37]. Specific mechanisms underlying the relationship between HCMV reactivation and GvHD are unclear and warrant further investigation. Alterations in T cell recovery, reduced thymopoiesis (observed in GvHD and HCMV reactivation) [37, 95, 96] or the induction of inflammatory cytokines associated with HCMV reactivation [97, 98] may contribute.

## 8 HCMV and Leukaemia Relapse

HCMV reactivation has been linked to protection from acute myeloid leukaemia (AML) relapse after allo‐HSCT in some studies [99, 100, 101, 102], although this does not translate to a survival advantage as HCMV reactivation is associated with increased non‐relapse mortality [103, 104]. Consistent evidence of an association between HCMV reactivation and AML relapse rates is lacking [51], and the effect may be restricted to specific patient subgroups varying with disease stage, conditioning regimen or use of T cell depletion [105, 106, 107]. A reduced incidence of relapse associated with early HCMV reactivation has been observed in recipients who are homozygous for HLA‐C killer cell immunoglobulin‐like receptor ligands groups 1 and 2, but not in heterozygous recipients (who have a higher proportion of licensed NK cells) [108], providing support for the hypothesis that HCMV‐induced NK cell populations are involved in mediating an anti‐leukaemic effect post‐HSCT [104, 109].

## 9 Immune Reconstitution Dynamics and Control of HCMV Reactivation in Allo‐HSCT

**FIGURE 2:**
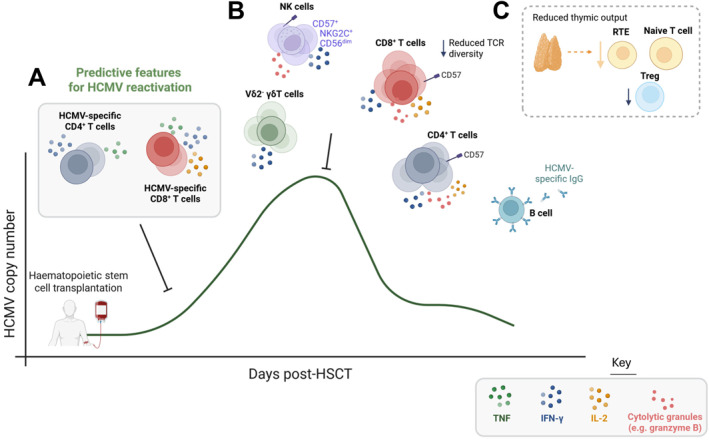
Features of immune reconstitution observed in allo‐HSCT recipients with HCMV reactivation. (**A**) Low frequencies of human cytomegalovirus (HCMV)‐specific CD4^+^ and CD8^+^ T cells at early timepoints (e.g., day 30 post‐transplant) predict the development of clinically significant HCMV infection in multiple studies. These HCMV‐specific T cells are often polyfunctional, expressing multiple cytokines in response to ex vivo stimulation with HCMV antigens. Expansion of functional HCMV‐specific T cell responses correlates with control of HCMV reactivation. (**B**) Memory/terminally differentiated CD8^+^ T cells and CD4^+^ T cells expressing CD57 and cytolytic molecules expand in allo‐HSCT recipients with HCMV reactivation, and T cell clonal expansions in HCMV infection correspond with reduced T cell receptor (TCR) diversity. Expansion of ‘adaptive’ NKG2C^+^ CD56^dim^ natural killer (NK) cells expressing CD57 and Vδ2^−^ γδ T cells has been linked to HCMV control, while the relative contribution of antibody responses to the control of HCMV reactivation is less clear. (**C**) Lower recovery of naïve T cells, recent thymic emigrants (RTE), and regulatory T cells (Treg) may suggest reduced thymic output in allo‐HSCT recipients who experience HCMV reactivation. HSCT, haematopoietic stem cell transplant; TNF, tumor necrosis factor; IFN‐γ, interferon gamma; IL‐2, interleukin 2. Created in BioRender. Stern, L. (2026) https://BioRender.com/148z13n.

**FIGURE 3:**
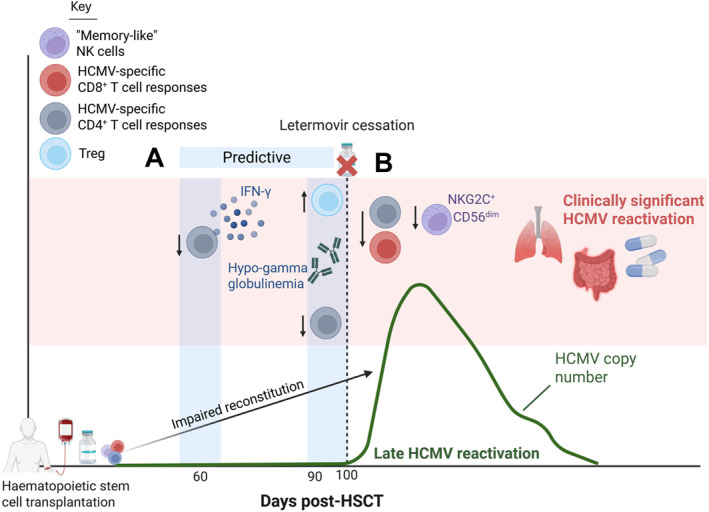
Immune reconstitution features reported to correlate with clinically significant HCMV infection in recipients of Letermovir prophylaxis. (**A**) Lower levels of IFN‐γ^+^ HCMV‐specific CD4^+^ T cells before letermovir cessation at day 60 post‐transplant may predict the development of clinically significant HCMV infection [132]. HCMV‐specific CD4^+^ T cell responses are also lower at day 90 in patients with clinically significant HCMV infection compared to controllers [117]. Higher regulatory T cell (Treg) frequencies at day 90 post‐transplant [132] and hypogammaglobulinemia (IgG < 650 mg/dL) at letermovir cessation [133] are associated with HCMV complications in letermovir recipients. **(B)** Poor HCMV‐specific CD4^+^ and CD8^+^ T cell recovery, and lower ‘memory‐like’ NKG2C^+^ CD56^dim^ natural killer (NK) cell counts, at later timepoints (days 180+) are evident in letermovir prophylaxis recipients who develop clinically significant HCMV infection, compared to controllers [117]. Human cytomegalovirus (HCMV), haematopoietic stem cell transplant (HSCT), IFN‐γ (interferon gamma). Created in BioRender. Stern, L. (2026) https://BioRender.com/29qma86.

Immune reconstitution dynamics are closely linked with HCMV reactivation and clinical outcomes in allo‐HSCT recipients. Both CD8^+^ and CD4^+^ T cells are of particular importance to the control of HCMV replication, and expand in response to HCMV reactivation [66, 68, 110, 111, 112, 113, 114]. Multifunctional HCMV‐specific T cells expressing cytokines such as interferon‐γ (IFN‐γ) and interleukin‐2 (IL‐2) are associated with protection from HCMV reactivation and enhanced viral control [68, 73, 115, 116] (Figure 2). The same is true in the letermovir era, where csHCMVi correlates with reduced IFN‐γ^+^ HCMV‐specific CD4^+^ and CD8^+^ T cell frequencies, as well as lower NKG2C^+^ CD56^dim^ NK cell recovery [117, 118] (Figure 3).

Higher frequencies of HCMV‐reactive T cell receptor (TCR) clones and reduced TCR diversity, particularly in the CD8^+^ T cell compartment, are evident in allo‐HSCT recipients with a HCMV seropositive recipient/donor (D+/R+) and those who have experienced HCMV reactivation [95, 119, 120, 121]. TCR sequencing found that HCMV‐reactive TCR‐α clonotypes made up to half of the CD8^+^ T cell repertoire in HCMV‐seropositive HSCT recipients [122]. Further evidence for a lasting immunologic imprint by HCMV is the prolonged expansion of CD57^+^ CD4^+^ effector memory T cells which occurs in HSCT recipients alongside HCMV exposure, particularly in D+/R− transplants [123]. Together with large expansions of effector memory and terminally differentiated CD4^+^ and CD8^+^ T cell populations expressing cytolytic molecules such as granzyme B [95, 124, 125], lower frequencies of recent thymic emigrants, naïve T cells and regulatory T cells (Tregs) have been observed in individuals who experience HCMV reactivation, suggestive of impaired thymic function [37, 95] (Figure 2).

HCMV infection directs NK cell differentiation towards more mature phenotypes [119, 126], and delayed NK cell reconstitution also correlates with increased HCMV reactivation in haploidentical HSCT recipients [127]. ‘Memory‐like’ CD56^dim^ NKG2C^+^ CD57^+^ NK cells with adaptive characteristics expand in response to HCMV reactivation and have been implicated in viral control and protection from leukaemia relapse [109, 128, 129, 130]. In patients with HCMV infection, the T cell depletion status of the graft modulates NK cell reconstitution, whereby NKG2C^+^ and CD56^neg^ NK cells with enhanced degranulation are more frequent in recipients of T cell depleted than T cell‐replete or double umbilical cord blood allografts [131]. These NKG2C^+^ NK cells only commence expansion after T cell recovery begins, potentially indicative of developmental cooperation between these two cell types [131].

### 9.1 Letermovir Prophylaxis

The introduction of letermovir prophylaxis has resulted in differences in immune reconstitution in comparison to that in patients without prophylaxis or receiving pre‐emptive therapy. It is thought this is likely due to prolonged suppression of HCMV replication. Higher NK cell counts and memory‐like NK cell frequencies, but lower CD8^+^ T cell frequencies across 30–120 days post‐transplantation, have been observed in recipients of letermovir prophylaxis compared to recipients of pre‐emptive therapy [132]. Low HCMV‐specific CD4^+^ T cell frequencies at 60 days post‐transplant, and higher Treg frequencies 90 days post‐transplant predicted persistent and symptomatic HCMV infection in letermovir prophylaxis recipients [132] (Figure 3). Hypogammaglobulinemia (IgG < 650 mg/dL) at letermovir cessation has also been indicated as a predictor of csHCMVi [133].

Immune reconstitution following HSCT can also be altered by PTCy for GvHD prophylaxis, which correlates with increased early HCMV reactivation [18, 93] and delayed CD4^+^ T cell recovery, but faster B cell reconstitution [92]. Similar HCMV‐specific CD8^+^ and CD4^+^ T cell reconstitution kinetics have been observed in haploidentical HSCT with PTCy compared to HLA‐matched allo‐HSCT [134].

### 9.2 Humoral Immunity

There is no consistent evidence for a relationship between HCMV antibody titres/neutralising antibody responses and HCMV control in allo‐HSCT recipients [135, 136, 137, 138]. There is also mixed evidence supporting the efficacy of pooled intravenous immunoglobulin (IVIG) or HCMV hyperimmune globulin in preventing HCMV reactivation, disease and mortality after HSCT [139, 140, 141, 142, 143]. Two randomised placebo‐controlled trials of monoclonal antibodies targeting HCMV glycoproteins failed to show a benefit in reduction of HCMV related events in the first few months post‐transplant [144, 145]. However, in a preclinical bone marrow transplant model, administration of strain‐specific antibodies could prevent murine cytomegalovirus (MCMV) reactivation [146]. Infusion of plasma containing likely HCMV strain‐specific antibodies also correlated with clearance of refractory HCMV infection in a case report [147]. Serum IgG levels < 400 mg/dL at day 100 post‐HSCT have been linked with an increased incidence of csHCMVi [148], and in a small cohort of umbilical cord blood transplant recipients, poor B cell recovery was observed in patients with uncontrolled HCMV infection [149].

### 9.3 Unconventional T Cells

Unconventional T cells include γδ T cells, mucosal‐associated invariant T (MAIT) cells and invariant NKT (iNKT) cells. Rapid reconstitution of γδ T cells occurs after HSCT, with HCMV able to shape the γδ TCR repertoire [150]. Vδ2^−^ γδ T cells have been seen to expand coincident with HCMV reactivation, and are capable of cross‐recognising HCMV infected cells and primary leukaemic blasts, producing IFN‐γ and mediating lysis of HCMV‐infected target cells [151, 152, 153]. Vδ2^+^ γδ T cells appear to be similarly endowed with protective antiviral features according to their cytotoxicity and IFN‐γ production in children receiving TCR αβ/CD19 cell depleted haploidentical HSCT [154]. Early recovery of γδ T cells decreases the risk of HCMV reactivation [155] indicating a potentially protective role in controlling HCMV viraemia after HSCT. CD8^+^ γδ T cells display increased expression of the protein Fc receptor‐like 6 (FCRL6) in individuals with HCMV reactivation [156].

Further studies are needed to understand the involvement of other innate‐like T cell populations such as MAIT and iNKT cells in HCMV infection post‐HSCT. A broad immune profiling approach using mass cytometry identified that peripheral blood MAIT cells were more frequent at the initial detection of HCMV reactivation in adult HSCT recipients with low‐level HCMV DNAemia, compared to those who developed high viral loads [125], and might therefore serve as a biomarker to identify individuals who can suppress reactivation without antiviral intervention. In children and young adults, a higher MAIT cell count at day 30 post‐HSCT was associated with increased HCMV reactivation [157]. HSCT patients receiving allografts with more iNKT cells have a lower incidence of HCMV reactivation [158].

### 9.4 Immune Monitoring and Biomarkers for HCMV

Biomarkers for the identification of HSCT recipients at risk of csHCMVi, or for stratification of HCMV infection severity, are essential to inform treatment intervention and limit unnecessary antiviral exposure. In seropositive recipients, poor CD4^+^ T cell recovery at day 30 post‐transplant has been associated with increased risk of developing high peak viral loads [159]. Monitoring the recovery of HCMV‐specific cell‐mediated immunity after transplantation is in current focus, as delayed recovery of HCMV‐specific CD4^+^ and CD8^+^ T cell responses correlates with increased risk of high‐level HCMV viraemia, recurrent viraemia or end‐organ disease [67, 69, 70, 71, 72, 73, 74, 75]. Approaches to measure HCMV‐specific cell‐mediated immunity in allo‐HSCT recipients include intracellular cytokine staining and flow cytometry [73, 115], tetramer staining [160, 161, 162], and commercially available assays such as the enzyme‐linked immunosorbent assay (ELISA) based QuantiFERON‐CMV assay [71, 72], and enzyme‐linked immunospot (ELISpot) based T‐SPOT.CMV assay [70, 163, 164] and T‐Track CMV assay [69, 165].

The QuantiFERON‐CMV assay measures IFN‐γ produced by HCMV‐reactive CD8^+^ T cells, where higher IFN‐γ production is associated with protection from HCMV disease and high viral loads [71, 72]. As a similar measure of HCMV‐specific cell‐mediated immunity, the ELISpot assays T‐SPOT.CMV and T‐Track CMV quantify IFN‐γ secretion after ex vivo stimulation of PBMCs with HCMV IE‐1 and pp65 peptides (T‐SPOT.CMV) or proteins (T‐Track CMV) that target both CD4^+^ and CD8^+^ T cells, and can allow identification of HSCT recipients expected to experience csHCMVi [70, 163, 165, 166]. A negative T‐Track CMV assay (absent HCMV‐specific immunity) demonstrated a high positive predictive value for HCMV related adverse events when used to predict recurrent HCMV after the first episode of HCMV reactivation or late recurrent HCMV after day 100 [69].

Recent assessment of the predictive success of QuantiFERON‐CMV and T‐SPOT.CMV found that they produced only moderately similar outcomes and despite high negative predictive value, they exhibited low positive predictive value, resulting in some patients with subsequent clinically significant disease going undetected [167]. Standardised monitoring of HCMV‐specific cell‐mediated immunity in allo‐HSCT is not currently part of routine clinical practice, but with future validation in randomised interventional clinical trials and definition of quantitative cut‐off thresholds, has the potential to inform tailored decisions on antiviral treatment, when combined with other clinical parameters including viral load monitoring [168, 169].

## 10 HCMV in Recipients of CAR T Cells

### 10.1 Intro to CAR T for ID Physicians

Chimeric antigen receptor T cells (CAR T) are novel cellular therapeutics comprising T cells gene modified to express an artificial antigen targeting receptor conjugated with an intracellular signalling domain that redirects T cell activity toward the target antigen [170]. Recipients of CAR T cells represent a unique new group of immunocompromised hosts and have distinct patterns of vulnerability to opportunistic pathogens. At the current time a small number of autologous CAR T products are available commercially, limited to haematologic malignancies, but is predicted to grow substantially with numerous CAR T therapeutics in development for non‐haematological malignancies, autoimmune and infectious diseases [171, 172, 173]. Other cellular products such as NK cells, allogeneic donor cells rather than autologous, or alternative gene modification strategies such as T cell receptors rather than CARs, are in development [174].

The kinetics of immune disturbance and the natural history of viral reactivation and tissue infection in CAR T recipients are less well characterised than for allogeneic transplant and will be the subject of ongoing research. This section of the review will focus on autologous CAR T for haematological malignancies, for which there is the largest body of clinical evidence and global distribution in routine clinical settings.

### 10.2 Treatment Journey

CAR T are generally administered as a salvage regimen after failure of other lines of therapy and the degree of immune suppression can be significant at the commencement of CAR T treatment. Lymphodepleting chemotherapy (fludarabine and cyclophosphamide, or bendamustine as single agent) is administered prior to CAR T infusion to enhance CAR T expansion and by design worsens the deficit of cellular immune function. As the CAR T are activated and expand in vivo, cytokine induced toxicities such as CRS and immune associated neurotoxicity syndrome (ICANS) can occur, for which further immune suppression with agents such as corticosteroids, tocilizumab and anakinra are administered, exacerbating vulnerabilities to infection [175]. These complications are largely restricted to the first 4 weeks post infusion. Later toxicities of hypogammaglobulinaemia from depletion of the CD19 or BCMA expressing compartment and immune effector cell associated haematological toxicity (ICAHT) can persist for months or years [176]. Overall, this combination of factors results in profound impairment to the cellular and innate immune response such that infection is the most common cause of non‐relapse mortality in these patients [177].

### 10.3 Impact on Immune Function

The status and trajectory of immune impairment and recovery depends on the number of lines of prior treatment, the intensity of lymphodepletion, the CAR construct (the target antigen and/or costimulatory domain), and the post‐CAR T toxicity and its management. Few studies have investigated cellular immune reconstitution kinetics. At the current time, all commercially available CAR T target the B cell lineage (CD19 expressing B cells or BCMA expressing plasma cells), but there is a growing body of clinical trial evidence using CARs targeting T cell antigens such as CD5 or CD7 demonstrating a very high rate of infection likely due to profound disruption of cellular immunity [178]. B cell aplasia and hypogammaglobulinaemia are very common complications of CD19 and BCMA directed CARs but it is unclear how much they contribute to HCMV risk. CD4 lymphopenia can be prolonged; 60% of patients had a CD4 count < 200 cells/μL at 12 months in one study [179]. HCMV specific immune function has been shown to fall to the nadir at 2 weeks but recover by 4 weeks [180], much faster than that seen in allogeneic transplant recipients.

### 10.4 Incidence and Natural History of HCMV in CAR T Recipients

The incidence of HCMV viraemia in recipients of CAR T varies substantially between studies but has been reported as high as 56% [181, 182, 183, 184, 185, 186, 187]. Clinically significant HCMV infection (defined as viraemia or end‐organ disease that required antiviral treatment) occurred in up to 15% [183, 184, 186]. HCMV tissue disease is infrequently seen (0%–3%) [182, 184, 185]. Involved organs include the colon and retina as in other immunocompromised populations. Encephalitis has been reported [188] but appears to be rare [189].

The pivotal studies of CAR T did not mandate HCMV surveillance, nor was detail provided in published results in many cases. When reported, incidence was generally in the range of 0%–3% [190, 191] which has since been shown to be an underestimate in real world and prospective studies. Retrospective studies are also likely to have underestimated the incidence of viral infections due to inconsistent viraemia monitoring, and the rate of csHCMVi may be overestimated due to under‐recognition of asymptomatic viraemia. In contrast, prospective studies have shown higher rates of viraemia. For example, a study of 72 HCMV seropositive CAR T recipients had a HCMV reactivation rate of 25% [180], in another of 51 patients, reactivation occurred in 56% after CAR T infusion [181]. The influence of early post‐CAR T toxicities and use of immune suppression is reflected in the median day of first reactivation (14–22 days) [180, 181, 183], corresponding to the nadir of cell mediated immunity.

There have been few studies of the natural history as pre‐emptive therapy has been instituted inconsistently. In some cases, thresholds similar to those used in the setting of allogeneic transplant have been applied, but it is unclear whether these relatively low thresholds are necessary to prevent the development of tissue infection [180, 192, 193]. Solano de la Asuncion et al. used a protocolised weekly surveillance strategy and higher treatment threshold (≥ 1500 IU/ml) to estimate the incidence of progression to tissue disease [181]. 24% of patients had viraemia at baseline and the cumulative incidence of HCMV reactivation was 57%. None of 51 patients developed tissue disease [181].

### 10.5 Risk Factors and Impact on Overall Outcomes

Factors that are known to impact cellular immune function have been confirmed as risk factors for HCMV in CAR T recipients, including prior HSCT, CRS/ICANS and use of immune suppressive agents. Four studies of HCMV reactivation have been collated in a systematic review [194]. Of 462 recipients of commercial and investigational CAR T (CD19, BCMA directed) HCMV reactivation occurred in 24.7% overall [194]. Risk was increased for recipients of BCMA CAR versus those who received CD19 CARs and with use of corticosteroid, tocilizumab or anakinra, more than 2 immune suppressants or longer exposure to corticosteroids (> 3 days) [180, 182, 183, 194]. The use of these agents is higher in the presence of higher grade of CRS or ICANS which was also associated with increased risk of HCMV viraemia as would be expected. Use of axicabtagene ciloleucel was associated with a higher rate of HCMV reactivation than tisagenlecleucel in one study [181].

HCMV reactivation is also associated with increased risk of poor outcomes [182, 183]. An increased overall mortality (53%–57% vs. 23%–38% at one year), non‐relapse mortality (48 vs. 33%) and lymphoma‐specific mortality (64 vs. 22%) is seen in HCMV reactivators versus non‐reactivators [182, 183]. The available data does not allow conclusions to be drawn about how much HCMV itself contributes to these outcomes or whether they reflect confounders such as high disease risk. Prospective studies should be performed to understand the contribution of disease, patient factors and CAR T products to the trajectory of CAR T associated HCMV infection.

### 10.6 Future Directions

Consensus about the definitions of HCMV‐related events are required to inform generalisability of future research findings. The definition of csHCMVi that has been adopted from transplant studies is problematic as it includes HCMV requiring treatment, as well as confirmed tissue disease [30]. With more data the threshold for intervention in CAR T recipients can be applied more consistently.

It is likely that the adverse outcomes that increase risk of HCMV reactivation are confounded by disease factors such that patients of higher disease risk who have higher risk of toxicity and therefore greater exposure to immune suppressants post CAR T are in turn at greater risk of viral reactivation. Likewise, the toxicity profile of different CAR T products may contribute as it influences the use of post‐CAR T use of immune suppression. Prospective multicentre studies should explore the contribution of these factors to determine the contribution, if any, of HCMV directly on long term outcomes. Correlative studies of immune function and viral dynamics should add to the understanding of the biological basis for reactivation to facilitate improved clinical interventions. Development and prospective evaluation of risk scores and practice guidelines will assist in identifying the subgroups at most risk, and guide appropriate management.

Emerging data on immune effects of novel CAR T constructs will increase complexity in the field. CARs targeting T cell antigens such as CD5 and CD7 have a profound effect on T cell number and function [195, 196]. Multi‐antigen CAR T, alternative cell types, CAR T with payloads such as co‐expression of cytokines will each have their unique signatures. It is expected that the lessons learnt from CARs directed toward the B cell lineage will be applied and greater detail will be provided about risk of viral infection from clinical trials of novel CAR T products, and as these enter clinical practice. In the long term, reduction in the rate of CRS and ICANS with the next generation of B cell directed CAR T products [197, 198] represents an opportunity to alleviate the associated immune suppression and infection risk.

## 11 Conclusion

HCMV reactivation remains a significant complication in transplant recipients, carrying a substantial healthcare burden, and is also associated with poor outcomes in recipients of CAR T therapy. There is a need to better understand the underlying basis of how HCMV impacts clinical outcomes in allo‐HSCT patients, and how standardised immune monitoring following transplantation could be implemented to allow for personalised antiviral management and treatment interventions. The significance of HCMV reactivation in individuals receiving CAR T therapy also deserves further evaluation in prospective studies. The landscape of HCMV reactivation and treatment in allo‐HSCT is currently evolving with the development of new prophylactic and therapeutic approaches. This includes the increasing application of letermovir prophylaxis in seropositive recipients, and clinical trials of virus‐specific adoptive T cell therapies for HCMV infection, which are discussed in Part 2 of this review.

## Author Contributions

LS, AA, BS and EB conceptualised the manuscript. DK, EB and LS assembled the initial draft. All authors reviewed and approved the manuscript.

## Funding

This work was supported by NHMRC Ideas Grant 2037493 awarded to BS and EB and NHMRC Ideas Grant 2019871 awarded to BS and AA, and a Translational Program Grant from the Cancer Council NSW, a grant from the Leukemia Foundation of Australia and a Centre for Research Excellence grant from the NHMRC awarded to DJG.

## Conflicts of Interest

EB holds patents in adoptive cell therapy for opportunistic infection and malignancy. EB reports advisory board membership for IQVIA, AbbVie, MSD, Astellas, Novartis, Gilead, and Bristol Myers Squibb, and research funding from MSD.

## Data Availability

Data sharing not applicable to this article as no datasets were generated or analysed in this study.
